# The influence of natural environment in residential areas on subjective well-being of the elderly

**DOI:** 10.3389/fpubh.2023.1037819

**Published:** 2023-03-13

**Authors:** Zhen Li, Yi Jiang, Ziyang Jin, Yiran Pu, Hao Li

**Affiliations:** ^1^School of Public Health and Management, Chongqing Medical University, Chongqing, China; ^2^Center for Public Health and Safety, Chongqing Medical University, Chongqing, China

**Keywords:** natural environment in residential areas, subjective wellbeing of the elderly, mediation effect, elderly's evaluation of the government's environmental protection work, Sobel test

## Abstract

**Objectives:**

The aim of this study is to explore the relationship between the natural environment of residential areas and the subjective wellbeing of the elderly and the role of elderly's evaluation of the government's environmental protection work in both.

**Methods:**

Based on the China Social Survey Database in 2013, 2015, 2017, 2019, Stata were used to process the data screened according to the restricted conditions. Ordered Probit Model and Sobel were used to test the effect relationship among the variables.

**Results:**

The subjective wellbeing of the elderly is roughly increasing. The natural environment of the living area has a significant positive effect on the subjective wellbeing of the elderly. The evaluation of the elderly on the government's environmental protection work has a positive impact on the elderly's subjective wellbeing similarly and plays an increasingly important intermediary role in the impact of the natural environment in the residential area on the elderly's subjective wellbeing.

**Conclusion:**

To improve the subjective wellbeing of the elderly, the government should continue to play a leading role in coordinating environmental protection and pollution control, strengthen publicity of environmental protection work. Moreover, improve the residential environment governance and protection system oriented by the elderly's evaluation of the government's environmental protection work.

## 1. Introduction

Subjective wellbeing (Abbreviated as SWB) results from evaluating one's quality of life according to the standards set by oneself (1). The SWB of the elderly reflects the overall spiritual life of the elderly and is one of the important indicators for measuring the physical and mental health and quality of life of the elderly (2). With the increasing proportion of the elderly population in China, the wellbeing of the elderly has become a more critical livelihood issue. The 14^th^ Five-Year Plan for the Development of the National Aging Cause and the Elderly Care Service System points out that the whole society should actively cope with the aging population pattern as it initially takes shape, and the elderly's sense of gain, happiness and security should be significantly improved.

At present, academics are focused on the definition and characteristics (3), the current situation and influencing factors (4–6), and practical research tools (7, 8) relating to the SWB of the elderly. Among them, the influencing factors are divided into subjective factors, such as personality traits and cognitive patterns, and objective factors, such as demographic factors, economic conditions, health conditions, and family life (7). However, the relationship between the natural environment and the SWB of the elderly is less discussed. China's environmental pollution has become a nuisance to residents' healthy lives, and the physical and psychological damage to residents cannot be ignored (9). According to the World Health Organization report, China ranked second worldwide in 2016 for the number of deaths caused by environmental pollution (10). Meanwhile, as people grow older, their bodily functions decline, as do their immunity and resistance. They are more vulnerable to be affected by external environmental pollution, thus inducing various diseases, and their SWB is more likely to be affected (11).

Second, most studies measure the quality of the natural environment in residential areas based on objective measurement data. For example, air pollution is calculated using the total amount of soot and dust emissions, nitrogen oxide emissions, and industrial waste gas in the past 12 months (12) or represented using the PM10 (Refers to particulate matter with particle size below 10 microns) level (13); the total amount of wastewater emissions from the whole society over the past 12 months is used to estimate water pollution (12); domestic waste pollution is estimated from the total amount of domestic waste collected and transported to disposal plants or sites in the past 12 months (12); the sound level of wind turbine noise in wind power generation is used to study its influence on residents' happiness (14). Few studies have been conducted from the perspective of the elderly's subjective cognition of the natural environment in the living area. In addition, the mediation effect of the elderly's evaluation of the government's environmental protection work on the living natural environment and the SWB of the elderly remains to be further studied in China. Based on the literature and social reality, this study speculated that elderly people had slightly different effects on their SWB from the living natural environment conditions depending on their evaluation of the government's environmental protection work.

Because of this, in combination with the China Social Survey (Abbreviated as CSS) database, this study uses Stata to try to explore the relationship between the living natural environment conditions of the elderly, their evaluation of the government's environmental protection work and their SWB, so as to provide a specific mechanism to formulate more targeted environmental governance policies for relevant government departments and provide theoretical support and policy ideas for helping “active aging.”

## 2. Literature review

### 2.1. The natural environment of the living area and the SWB of the elderly

In the 1980's, Tao's “human settlement theory” was translated by Wu Liangyong, a famous Chinese scholar, and developed into a new disciplinary system of human settlement science (15), which focuses on the coordination between man and nature and focuses on the living environment. The five principles of human settlement environment construction indicate that we should face the ecological dilemma squarely and improve our ecological consciousness, care for all people, and attach importance to the overall interests of social development. In recent years, China has made active efforts to control environmental pollution, but environmental pollution is still a major threat to the construction of a better living environment and has become an important reason for the deterioration of Chinese people's wellbeing (16).

The natural environment of residential areas includes various elements, such as air and water, and research (12) has depicted that public awareness is strongest around the three environmental pollution problems of water pollution, air pollution, and household waste pollution. Many scholars have discussed the impact of air pollution on SWB. According to the 2005–2018 Gallup World Poll of 151 countries, better subjective cognition of air quality is associated with higher personal happiness (17). Subjective cognition of air pollution has a more serious impact on happiness than Objective cognition of air pollution (17). Air pollution has a more significant negative impact on the wellbeing of unhealthy and elderly people than healthy people and young people (18). In addition, studies have found that air pollution negatively correlates with people's ethical behavior and that air pollution exacerbates anxiety, which in turn may increase unethical behavior (19). This means that the interactions between air pollution and SWB need to be further studied, and certain moderating and mediating variables may explain the relationship between air pollution and SWB. Like air, water quality is closely related to the SWB of the elderly. Studies (20) have demonstrated a correlation between residents' mental health and perceived water pollution in China, Japan, and South Korea. Furthermore, psychological and mental health issues like anxiety and depression are also related with exposure to noise pollution (21). Due to the influence of airport noise, the happiness of residents living within 50 km of Amsterdam Airport is significantly lower than that of those living over 50 km away (22). In addition, other environmental pollution (such as land pollution and electromagnetic ionizing radiation pollution) is strongly correlated with the SWB of the elderly (23). According to a report from the World Health Organization, electromagnetic radiation pollution has become the fourth major environmental pollution after air, water, and noise pollution. Residents exposed to soil pollution have poorer psychosocial health than those who are not exposed, with higher levels of anxiety and depression (24). Based on this, the following hypothesis is put forward:

Hypothesis 1: The natural environment of the residential area has a positive impact on the SWB of the elderly. The better the natural environment of the residential area, the higher the SWB of the elderly. On the contrary, the worse the natural environment of the residential area, the lower the SWB of the elderly.

### 2.2. The intermediary role of the elderly in the evaluation of the government's environmental protection work

On the one hand, both objective environmental quality and residents' subjective perception of environmental quality will impact the government's environmental protection work evaluation. Studies (25) have depicted that the improvement of objective environmental quality does not necessarily improve residents' evaluation of the government's environmental work, on the contrary, it may reduce the correlation positive evaluation. However, the public's subjective perception of environmental pollution has a significant direct impact on the assessment of the government's environmental work. The more serious the perceived pollution, the lower the rating of the government's environmental work. On the other hand, uncertainty about the health effects of exposure to chronic and invisible pollutants can lead to distrust of policymakers by victims and conflict within communities (26). In environmental risk perception, the government's environmental behavior positively impacts residents' perception of the risks to their quality of life and mental health. For example, Song et al. (27) used panel data composed of microscopic data from China Family Panel Studies database (CFPS) 2012, 2014, and 2016 to conduct an empirical test and found that residents' SWB continuously improved with the enhancement of environmental regulations. And the enhancement of environmental regulations can indirectly improve the SWB of residents by improving residents' health status.

Hypothesis 2: The elderly's evaluation of the government's environmental protection work plays an intermediary role in the impact of natural environmental pollution on the elderly's SWB.

**Figure 1:**
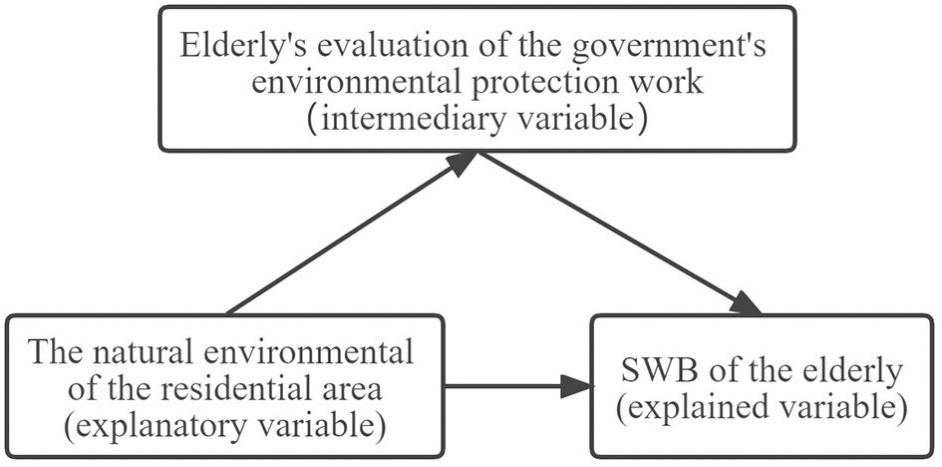
Theoretical model of the influence of natural environment in residential area on subjective well-being of the elderly.

As mentioned above, this study attempts to incorporate the natural environment in the residential area, elderly's assessment of the government's environmental protection work, and the elderly's SWB into the same analysis framework demonstrated in Figure 1.

## 3. Data source and methods

### 3.1. Data source

The data used in this study are from the CSS conducted by the Institute of Sociology of the Chinese Academy of Social Sciences. CSS is a biennial longitudinal survey that adopts the method of probability sampling. The survey area covers 31 provinces (autonomous regions/municipalities) directly under the central government, including 151 counties (districts) and 604 villages (neighborhood committees). Each survey visits more than 10,000 urban and rural families. The research results can infer the country's household population aged 18–69. In this study, owing to CSS is a biennial longitudinal survey, so samples respondents with age characteristics over 60 years old in CSS2013, CSS2015, CSS2017 and CSS2019 were selected from the CSS database, and the number of valid samples respondents was 1,801, 2,005, 2,248, and 1,049, respectively after eliminating the missing values.

### 3.2. Variable selection

The Explained variable in this study was the SWB of the elderly. In Economics of Happiness, Life satisfaction (LS) can be a proxy variable of happiness (28). Deaton and Stone (29) proposed that SWB is a cognitive process, which is related to an individual's overall evaluation of life, or the overall memory of an individual's living conditions for a period of time. The measurement method is based on Cantrell Ladder Method, which divides life satisfaction into 10 grades, representing satisfaction levels of different quality of life from low to high. In this study, the corresponding question in the CSS2013, CSS2015, CSS2017 and CSS2019 questionnaires “Please use 1–10 points to express your satisfaction with life, 1 point to express very dissatisfied, 10 points to express very satisfied” to express the SWB of the respondents. The higher the number, the higher the satisfaction with life. Studies have revealed that although this measurement method is simple, it also has high reliability and validity (30).

The explanatory variable was satisfaction with the environmental conditions of the residence (ES). In this study, the corresponding question in the CSS2013, CSS2015, CSS2017 and CSS2019 questionnaires, “Please use 1–10 points to express your satisfaction with the environmental conditions of your residence. 1 point means very dissatisfied, and 10 points means very satisfied” to express the respondents' satisfaction with the environmental conditions of their residence. The higher the number, the higher the respondents' satisfaction with the environmental conditions of their residence.

This study took the evaluation of the elderly on the government's environmental protection work (GV) as an intermediary variable. The corresponding question in the CSS2013, CSS2015, CSS2017 and CSS2019 questionnaires, “Is the government doing a good job in environmental protection and pollution control?” Eliminate the “not clear” option, and the alternative answers were “excellent,” “relatively good,” “not so well,” “terrible.”

Apart from the living environment's influence on SWB in older adults, personal characteristics are highly correlated with SWB. For example, social comparison theory reveals that happiness is influenced by comparing one's income or status with the average level of the whole society. In addition, other personal characteristics, such as education level and work status, similarly affect the subjective assessment by the elderly of their external living conditions and wellbeing (31). Therefore, demographic factors such as gender, education level, marital status, working status, political status, nature of household registration and personal annual income, which are control variables, were included in the study.

### 3.3. Methods

**Figure 2:**
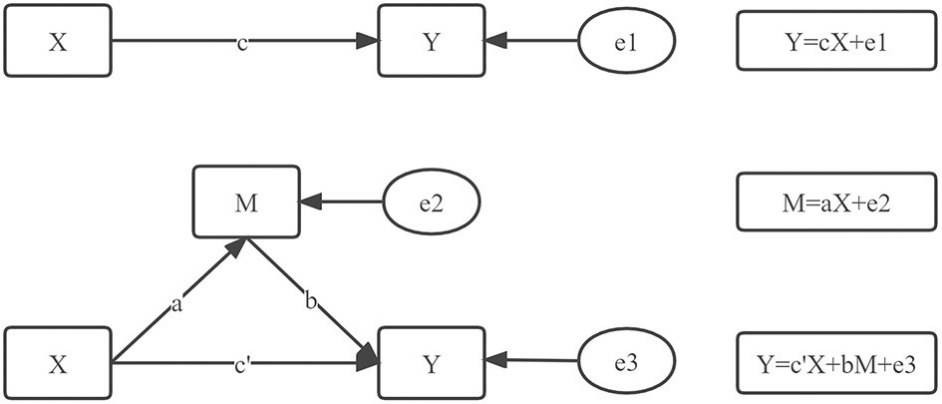
Regression equation and path diagram.

The life satisfaction of the elderly, the evaluation of the elderly on the government's environmental protection work, and satisfaction with the environmental conditions of the residence did not conform to the expected data. So Spearman correlation analysis and Ordered Probit Model were used to test the correlation and interaction of the main variables. The difference was statistically significant with *P* < 0.05. The intermediary effect test model of Wen and Ye (32) was used to examine the intermediary effect of the elderly on evaluating the government's environmental protection work. As shown in Figure 2, coefficient *c* represented the total effect obtained through the regression analysis of the independent variable *X* and the dependent variable *Y*; Coefficients *a* and *b* represented intermediate effect process values. *a* was obtained by regression analysis of independent variable *X* and intermediary variable *M*. *b* was obtained by regression analysis of intermediary variable *M* and dependent variable *Y* after controlling the influence of independent variable *X*. The coefficient *c*' represented the direct effect value. After controlling the influence of the intermediary variable *M*, it was obtained through the regression analysis of the independent variable *X* on the dependent variable *Y*. *e*1, *e*2, and *e*3 represented the regression residual.

## 4. Results

### 4.1. Descriptive statistical analysis

As shown in Table 1, from 2013 to 2019, almost 90% of the sample respondents' life satisfaction was “5” and above in each year, indicating that the respondents were relatively satisfied with their lives. The sample respondents' satisfaction with the living natural environment was “8,” which accounted for the largest proportion in each year. The proportion of the sample respondents who thought that the government's environmental protection work was “Not so well” and dissatisfied with the government's environmental protection work fell from 35.7% in 2013 to 19.2% in 2019.

**Table 1:** Descriptive statistics of main variables.

		**2013 (*****N*** = **1,801)**		**2015 (*****N*** = **2,005)**		**2017 (*****N*** = **2,248)**		**2019 (*****N*** = **1,049)**	
**Variables**	**Option**	**Frequency**	**Percentage (%)**	**Frequency**	**Percentage (%)**	**Frequency**	**Percentage (%)**	**Frequency**	**Percentage (%)**
LS	1	19	1.1	33	1.7	53	2.4	24	3.0
	2	22	1.2	34	1.7	45	2.0	19	1.8
	3	36	2.0	77	3.8	88	3.9	27	2.6
	4	77	4.3	109	5.4	105	4.7	26	2.5
	5	248	13.8	396	19.8	387	17.2	128	12.2
	6	266	14.8	336	16.8	305	13.6	119	11.3
	7	308	17.1	313	15.6	282	12.5	120	11.4
	8	461	25.6	393	19.6	494	22.0	213	20.3
	9	175	9.7	161	8.0	168	7.5	87	8.3
	10	189	10.5	153	7.6	321	14.3	286	27.3
ES	1	38	2.1	87	4.3	135	6.0	46	4.4
	2	28	1.6	72	3.6	83	3.7	10	1.0
	3	57	3.2	111	5.5	136	6.1	31	3.0
	4	71	3.9	162	8.1	109	4.9	33	3.2
	5	215	11.9	369	18.4	391	17.4	178	17.0
	6	279	15.5	294	14.7	238	10.6	96	9.15
	7	313	17.4	271	13.5	269	12.0	129	12.3
	8	479	26.6	376	18.8	423	18.8	230	22.0
	9	164	9.1	118	5.9	157	7.0	64	6.1
	10	157	8.7	145	7.2	307	13.7	232	22.1
GV	Excellent	160	8.9	254	12.7	467	20.8	294	28.0
	Relatively good	999	55.5	1,063	53.0	1,120	49.8	554	52.8
	Not so well	522	29.0	528	26.3	468	20.8	152	14.5
	Terrible	120	6.7	160	8.0	193	8.6	49	4.7
Gender	Male	870	48.3	1,027	51.2	1,145	50.9	492	46.9
	Female	931	51.7	978	48.8	1,103	49.1	557	53.1
Education	Not in school	422	23.4	470	23.4	515	22.9	227	21.7
	Primary school	754	41.9	761	38.0	837	37.2	326	31.1
	Junior high school	398	22.1	476	23.7	561	25.0	290	27.7
	High school	157	8.7	217	10.8	254	11.3	169	16.1
	Junior college	52	2.9	50	2.5	50	2.2	30	2.9
	Bachelor degree	15	0.8	26	1.3	26	1.2	4	0.4
	Graduate students and above	3	0.2	5	0.3	5	0.2	3	0.3
Marital	Unmarried	25	1.4	23	1.2	32	1.4	16	1.5
	Married	1,751	97.2	1,955	97.5	2,186	97.2	1,017	97.0
	Divorced	25	1.4	27	1.4	30	1.3	16	1.5
Political	Party member	1,544	85.7	272	13.6	303	13.5	164	15.6
	Non-party people	257	14.3	1,733	86.4	1,945	86.5	885	84.4
Nature of household registration	Agricultural household	1,252	69.5	1,346	67.1	1,554	69.1	707	67.4
	Non-agricultural household	549	30.5	659	32.9	694	30.9	342	32.6
Job	Have a job	809	44.9	946	47.2	1,164	51.8	540	51.5
	No job	992	55.1	1,059	52.8	1,084	48.2	509	48.5
Annual income	-	8.8 (*m*)	3.0 (*SD*)	8.5 (*m*)	2.4 (*SD*)	8.0 (*m*)	3.1 (*SD*)	8.3 (*m*)	2.9 (*SD*)

N, sample size; m, mean; SD, standard deviation; Annual income: total personal income of the previous year after logarithm.

### 4.2. Spearman correlation analysis

It can be seen from Table 2 that the correlation coefficients between residents' satisfaction with environmental conditions and their life satisfaction of the sample respondents from 2013 to 2019 were 0.3, 0.5, 0.6 and 0.3, respectively, indicating that they were positively correlated and had statistical significance (*P* < 0.001), which was consistent with the research conclusions of Huang and He (33); In addition, the correlation coefficients between the evaluation of the sample respondents on the government's environmental protection work and their life satisfaction were −0.1 (*P* < 0.001), −0.1 (*P* < 0.001), −0.1 (*P* < 0.001), −0.2 (*P* < 0.001), respectively from 2013 to 2019, indicating that the two were negatively correlated; the correlation coefficients of the sample respondents' satisfaction with environmental conditions and their evaluation of the government's environmental protection work were −0.4 (*P* < 0.001), −0.3 (*P* < 0.001), −0.2 (*P* < 0.001), −0.4 (*P* < 0.001), respectively, indicating that they were also negatively correlated. In this study, owing to the indicators of the elderly's evaluation of the government's environmental protection work were reverse values, the larger the value, the lower the respondents' evaluation of the government's environmental protection work. Therefore, the sample respondents' evaluation of government environmental protection work was negatively related to their life satisfaction and their satisfaction with natural environmental conditions.

**Table 2:** Correlation analysis of main variables.

	**2013 (*****N*** =**1,801)**			**2015 (*****N*** = **2,005)**			**2017 (*****N*** = **2,248)**			**2019 (*****N*** = **1,049)**		
**Variables**	**LS**	**ES**	**GV**	**LS**	**ES**	**GV**	**LS**	**ES**	**GV**	**LS**	**ES**	**GV**
LS	1.0			1.0			1.0			1.0		
ES	0.3^***^	1.0		0.5^***^	1.0		0.6^***^	1.0		0.3^***^	1.0	
GV	−0.1^***^	−0.4^***^	1.0	−0.1^***^	−0.3^***^	1.0	−0.1^***^	−0.2^***^	1.0	−0.2^***^	−0.4^***^	1.0

N, sample size; LS, Life satisfaction; ES, Satisfaction with living natural environment; GV, Evaluation of government environmental protection work; ^*^P < 0.05, ^**^P < 0.01, ^***^P < 0.001.

### 4.3. Regression analysis

To further investigate the impact of the quality of the natural environment in residential areas on the SWB of elderly, this study adopted Ordered Probit Model, including three models. It can be seen from Table 3 that in the 4 years, the satisfaction of the sample respondents with the living natural environment has been positively and significantly influencing their life satisfaction (*P* < 0.001). Moreover, the *R* value of the model 1 in 2013 was 0.09, which means that the satisfaction of the residential environment can explain the 9% change of the life satisfaction. For model 2, after adding control variables on the basis of model 1, the *F* value changes significantly (*P* < 0.05), which means that the control variables has explanatory significance for the model after adding. For model 3, after adding the evaluation of the samples respondents on the government's environmental protection work on the basis of model 2, its impact on life satisfaction was not significant (*P* > 0.05), as was the case in 2015. The evaluation of the samples respondents on the government's environmental protection work was added on the basis of model 2 in 2017, and we found that it had a negative impact on life satisfaction, with a regression coefficient of −0.09 (*P* < 0.05). In 2019, the regression coefficient was −0.26 (*P* < 0.01), which was also statistically significant.

**Table 3:** Regression analysis of main variables.

	**LS**											
	**2013 (*****N*** = **1,801)**			**2015 (*****N*** = **2,005)**			**2017 (*****N*** = **2,248)**			**2019 (*****N*** = **1,049)**		
	**M 1**	**M 2**	**M 3**	**M 1**	**M 2**	**M 3**	**M 1**	**M 2**	**M 3**	**M 1**	**M 2**	**M 3**
ES	0.29^***^ (0.02)	0.31^***^ (0.02)	0.32^***^ (0.02)	0.48^***^ (0.02)	0.48^***^ (0.02)	0.48^***^ (0.02)	0.49^***^ (0.02)	0.49^***^ (0.02)	0.48^***^ (0.02)	0.28^***^ (0.03)	0.29^***^ (0.03)	0.26^***^ (0.03)
GV	-	-	−0.04 (0.06)	-	-	−0.04 (0.05)	-	-	−0.09^*^ (0.05)	-	-	−0.26^**^ (0.09)
Control	-	YES	YES	-	YES	YES	-	YES	YES	-	YES	YES
*F*	186.31	34.87	31.03	852.36	121.60	108.13	1,029.71	137.72	123.04	96.92	17.13	16.15
Prob > *F*	0.00	0.00	0.00	0.00	0.00	0.00	0.00	0.00	0.00	0.00	0.00	0.00
*R*	0.09	0.14	0.14	0.30	0.33	0.33	0.31	0.33	0.33	0.08	0.12	0.12

N, sample size; M, Model; LS, Life satisfaction; ES, Satisfaction with living natural environment; GV, Evaluation of government environmental protection work; Standard error in brackets; ^*^P < 0.05, ^**^P < 0.01, ^***^P < 0.001.

### 4.4. Robustness check

In order to verify the robustness of the results, we used the ordinary least squares (OLS) Model to estimate. The results are shown in Table 4. The probability of “very satisfied” life satisfaction of the samples respondents increased by 31.6, 48.3, 48, and 25.9%, respectively when their satisfaction with the living natural environment increased by one level in 2013, 2015, 2017 and 2019. In addition, the *D-W* value was close to 2, indicating that there is no autocorrelation in the model and no correlation between sample data, the model is good. The above regression results showed that whether demographic variables were controlled or different measurement methods were used, the satisfaction of the elderly's living environment had a significant positive impact on their SWB. Hypothesis 1 is valid.

**Table 4:** OLS regression analysis.

**Variables**	**2013 (*N* = 1,801)**	**2015 (*N* = 2,005)**	**2017 (*N* = 2,248)**	**2019 (*N* = 1,049)**
ES	0.316** (11.646)	0.483** (23.388)	0.480** (26.050)	0.259** (7.037)
GV	−0.042 (−0.631)	−0.037 (−0.682)	−0.094 (−1.786)	−0.258* (−2.514)
Gender	0.157 (1.693)	0.220** (2.654)	−0.049 (−0.581)	0.121 (0.806)
Education	0.058** (4.700)	0.031** (2.780)	0.012 (1.008)	0.019 (1.018)
Marital	−0.403 (−1.384)	0.385 (1.634)	−0.176 (−0.715)	−0.916* (−2.311)
Political	0.280* (2.364)	0.189 (1.907)	0.269** (2.634)	0.476** (3.314)
Nature of household registration	0.404** (3.649)	0.400** (3.841)	0.381** (3.505)	0.207 (1.166)
Job	0.051 (0.491)	0.124 (1.310)	0.020 (0.217)	−0.094 (−0.574)
Annual income	−0.002 (−0.120)	0.048* (2.486)	0.024 (1.664)	0.074** (2.790)
*R*-Square	0.135	0.328	0.331	0.123
Adjusted *R*-Square	0.131	0.325	0.328	0.115
*F*	*F* (9, 1, 791) = 24.050, *P* = 0.000	*F* (9, 1,995) = 83.543, *P* = 0.000	*F* (9, 2,238) = 93.559, *P* = 0.000	*F* (9, 1, 039) = 14.814, *P* = 0.000
*D-W*	1.733	1.905	1.954	2.012

N, Sample size; Explained variable: LS; ^*^P < 0.05 ^**^P < 0.01; t value in parentheses.

### 4.5. Intermediary effect test

According to the Ordered Probit Model analysis results, the evaluation of the samples respondents on the government's environmental protection work had a significant negative impact on their SWB in 2017 and 2019. This provided conditions for the next step to test whether the evaluation of the elderly on the government's environmental protection work had an intermediary effect. We used Sobel test (32) by Stata and found that the mediating effect of it in 2017 accounted for 1.19% (*P* < 0.05), and in 2019 rose to 11.12% (*P* < 0.05) (Table 5), indicating that it plays an increasingly important intermediary role in the impact of living natural environment conditions on the SWB of the elderly. Hypothesis 2 is verified.

**Table 5:** Mediating effect test.

	**2017**				**2019**			
	**Coef**	***SE***	***Z***	***P**>**|Z|***	**Coef**	***SE***	***Z***	***P**>**|Z|***
a	−0.062	0.007	−8.945	0	−0.126	0.009	−13.620	0
b	−0.094	0.046	−2.030	0.042	−0.258	0.094	−2.731	0.006
Indirect effect	0.006	0.003	1.98	0.048	0.032	0.012	2.677	0.007
Direct effect	0.480	0.015	31.273	0	0.259	0.031	8.491	0
Total effect	0.486	0.015	32.189	0	0.291	0.281	10.338	0
Proportion of total effect that is mediated	0.0119				0.1112			

## 5. Discussion

The natural environment of the living area has a significant positive effect on the SWB of the elderly. It is consistent with the conclusions of previous studies (34). The elderly's evaluation of the government's environmental protection work similarly has a significant positive impact on their SWB. The reason may be that environmental pollution has a direct and long-term harmful impact on human life and health, and the elderly worries about their health and panic about the living environment will stimulate their identification and discussion of the responsibility for environmental protection. According to principal-agent theory, the relationship between citizens and the government can be regarded as a principal-agent relationship (35). The government, as the trustee of public fiduciary social responsibility, should properly manage environmental and other public resources on behalf of citizens after obtaining public power. Although the government has actively taken many measures to improve environmental quality, the above results depicted that 19.4% of sample respondents were still unsatisfied with the government's environmental protection work. Environmental pollution has gradually surpassed itself and become a political and social issue. Therefore, the elderly's evaluation of the government's environmental protection work may profoundly impact the SWB of elderly people (36). In addition, the more serious elderly's subjective perception of the living environment pollution, the more frequently they discuss the environmental pollution with their families and friends, thus becoming suspicious and dissatisfied with the government's environmental protection work. Such social interactions often lead to the spread of negative emotions and further reduce the SWB of the elderly (37).

The elderly's evaluation of the government's environmental protection work plays an increasingly important intermediary role in the impact of living natural environment conditions on the SWB of the elderly. Satisfaction with the government's investment in environmental protection is the primary source of the elderly's subjective happiness perception of the living environment (38). The mechanism is that the government effectively solves the air, water, soil, and other pollution problems. On the one hand, this reduces the health problems caused by environmental pollution and the treatment costs of related diseases, healthy body and reduced spending significantly improve the SWB of the elderly. On the other hand, the government's environmental protection work improves the environmental quality, and the elderly experience the improvement of life quality through factors such as blue skies, white clouds, and clean water, which not only reduces their psychological pressure but also helps to enhance their trust in the government, thus enhancing their own SWB (39).

## 6. Conclusion

Based on the above discussions, we can see: The better the natural environment of the living area, the higher the SWB of the elderly; The better the evaluation of the government's environmental protection work by the elderly, the higher the SWB of the elderly; In addition, the natural environment of the living area gradually affects the SWB of the elderly by influencing their evaluation of the government's environmental protection work. Therefore, the corresponding conclusions are drawn: First, to improve the SWB of older people, the government should continue to coordinate environmental protection and pollution control. Such as taking “improving residents' perceived environmental quality” as part of its policy objectives, implement an environmental protection target responsibility system and strengthen the quantitative assessment of comprehensive remediation of the urban environment. Second, the government should increase the publicization of its environmental protection work in various ways so that the elderly can see the government's efforts to improve the quality of the living environment, which would improve the elderly's assessment of the government's environmental protection activities. For example, establish an open and transparent environmental information disclosure system, regularly publicize pollution level measurements, publish environmental quality improvement results in the media, and inform the public of progress toward achieving targets. Third, the government should strengthen communication with residents so that they can understand the residents' subjective perception of the living natural environment and demands in a timely manner and carry out the necessary follow-up work.

There are still deficiencies in the study. This study only verifies the impact of the single intermediary role of the elderly in evaluating government environmental protection work on the SWB of the elderly. Whether the SWB of the elderly will be affected by other intermediary variables needs to be further studied.

## Data availability statement

Publicly available datasets were analyzed in this study. This data can be found at: http://csqr.cass.cn/DataExplore/?ProjectID=2018061909463245927261066314. Repository: CSS2013, CSS2015, CSS2017, and CSS2019. Accession number: 18383022018.

## Author contributions

Methodology, data collection, and analysis were performed by ZL. The first draft of the manuscript was written by ZL, ZJ, and YJ. All authors commented on the previous versions of the manuscript, contributed to the study conception and design, read, and approved the final manuscript.
